# Why aren’t they used? Systematic review of barriers to implementation of clinical decision support systems for early cancer detection in primary care

**DOI:** 10.3399/BJGP.2025.0579

**Published:** 2026-02-24

**Authors:** Christina Derksen, Kate A Le Noury, Adriana B Akbar, Thomas Round, Fiona M Walter, Suzanne E Scott

**Affiliations:** 1 Wolfson Institute of Population Health, Faculty of Medicine and Dentistry, Queen Mary University of London, London, UK; 2 Barts and the London School of Medicine and Dentistry, Faculty of Medicine and Dentistry, Queen Mary University of London, London, UK; 3 School of Life Course and Population Sciences, King's College London, London, UK

**Keywords:** decision support systems, clinical, early detection of cancer, implementation science, primary health care

## Abstract

**Background:**

Early cancer detection is crucial for patient outcomes. Clinical decision support systems (CDSSs) have been developed to assist with decision making about screening or symptomatic assessment in primary care, but implementation remains challenging.

**Aim:**

To compare barriers to implementation of cancer-specific CDSSs for screening and symptomatic presentation in primary care.

**Design and setting:**

Systematic mixed-methods literature review.

**Method:**

A subanalysis within a systematic review was conducted. Qualitative and quantitative data on barriers were coded into themes guided by the Theoretical Domains Framework. Frequencies of studies mentioning barriers were compared between CDSSs for cancer detection and other conditions, and between cancer-specific CDSSs for screening and symptomatic presentation.

**Results:**

In total, 29 cancer-specific CDSSs were identified, addressing screening (*n* = 15) and symptomatic presentation (*n* = 14), with a further 70 addressing other conditions. There were minimal differences in barriers for cancer-specific CDSSs and other CDSSs. There were differences between cancer-specific CDSSs for screening and symptomatic presentation. Barriers more frequently reported for CDSSs for symptomatic presentation involved workflow integration (*n* = 9/14, 64% versus *n* = 4/15, 27%), medicolegal uncertainty (*n* = 4/14, 29% versus *n* = 0/15, 0%), requirements of skills (*n* = 7/14, 50% versus *n* = 2/15, 13%), interference with decision-making processes (*n* = 6/14, 43% versus *n* = 2/15, 13%), and negative emotions (*n* = 8/14, 57% versus *n* = 4/15, 27%).

**Conclusion:**

The function and healthcare context of CDSSs in the diagnostic process (symptomatic assessment or screening decision making) appears to be more relevant to implementation than the targeted condition. Involving stakeholders to clarify medicolegal issues and workflow integration is essential for the implementation of CDSSs for symptomatic presentation.

## How this fits in

Clinical decision support systems (CDSSs) are increasingly being developed to support screening and early diagnosis of cancer in primary care, but practitioners often face barriers when using these systems. This review summarises the evidence about why cancer-specific CDSSs have been underutilised and suggests that CDSSs for symptomatic presentation (predominantly used in the UK and Australia) are more difficult to implement than CDSSs for screening (predominantly used in the US). The findings underpin the importance of existing recommendations for clinicians using CDSSs, especially for symptomatic presentation. Recommendations include the clarification of medicolegal responsibilities and liability, dedicated training to improve communication with patients when systems raise cancer suspicions, and continued professional development regarding knowledge and management skills of cancer.

## Introduction

For most cancers, diagnosis at an early stage is crucial for timely treatment, better long-term outcomes, and reducing healthcare costs.^1,2^ There are better overall survival rates and longer median survival in patients diagnosed at earlier stages across breast cancer, lung cancer, melanoma, head and neck cancer, and bladder cancer.^3^ Earlier detection allows for less invasive treatment, substantially reducing medical care costs.^4^

Early detection can be facilitated by screening programmes.^5^ However, screening uptake is often lower than recommended.^6^ Despite the benefits, screening can cause harm, such as adverse procedures, overdiagnosis, and overtreatment in several cancer types.^7–9^ Therefore, strategies are needed to provide balanced information to enable informed choice,^10^ and this role often falls to primary care. Where there is no established screening programme, early diagnosis relies on timely symptomatic presentation to a healthcare professional and onward referral for investigation and diagnostic testing. Patients often present with non-specific symptoms that could be attributed to multiple conditions.^11,12^ Primary care practitioners (PCPs) have to consider differential diagnoses with different prevalence, comorbidities, and risk factors.^13^

Clinical decision support systems (CDSSs) have been developed to assist PCPs and patients, with current research focusing on the development of more accurate risk prediction models using new technology such as artificial intelligence (AI).^14^ Some CDSSs aim to facilitate decision making around cancer screening by balancing potential benefits for the individual with the potential risks of overdiagnosis (for example, Militello *et al*, Carney *et al*, and Lowery *et al*).^15–17^ Other CDSSs aim to support PCPs by providing risk assessments and alerts to support appropriate referrals for further investigation of symptomatic patients (for example, Akanuwe *et al*, Black *et al*, and Chima *et al*).^18–20^ The NHS 10-year health plan for England forecasts that AI algorithms will *‘detect subtle signs of disease years before symptoms appear, and help clinicians choose the most effective, personalised treatments.’* ^21^

A systematic review of effectiveness of CDSSs on diagnostic decision making found that CDSSs used during consultation did not have an effect on appropriateness of referral but did improve the quality of the referral and might be cost-effective despite low specificity.^22^ CDSSs used outside the consultation (such as creating a list of patients from the electronic health record for case finding) seemed more promising, showing a reduction in time to diagnostic evaluation or triggering further review.^23,24^

For such tools to have a positive impact, successful implementation is essential. Yet implementation of CDSSs into routine practice is a challenge. In a survey of UK practitioners, Price *et al* ^25^ found that, despite being available, cancer-specific CDSSs were used in only 17% of primary care practices. In their feasibility trial, Rubin *et al* ^26^ reported interoperability problems, restrictions with software installation, and a lack of fit with primary care workflows, preventing implementation of a CDSS for possible oesophago-gastric cancers.

**Figure 1.**
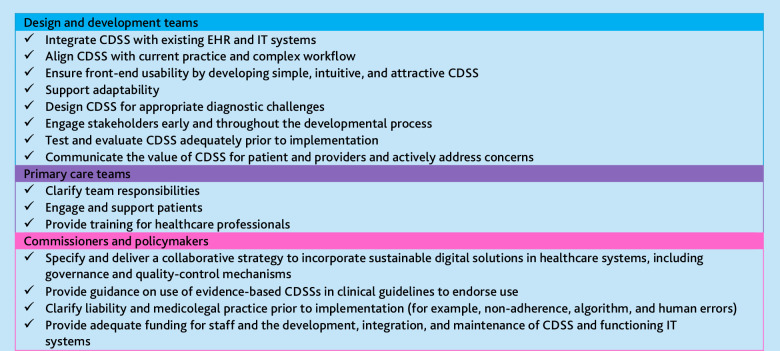
Recommendations for design and implementation of CDSSs in primary care, adapted from Derksen *et al.* ^27^ CDSS = clinical decision support system. EHR = electronic health record. (All details: https://www.candetect.org/research/workstream-3/recommendations-for-the-implementation-of-clinical-decision-support-systems)

In a recent systematic review, the current author group identified barriers to the implementation of CDSSs in primary care and a suite of recommendations for improving implementation.^27^ A wide range of interrelated barriers to implementation were identified, including workflow and workload barriers within primary care, IT systems, scepticism towards CDSSs, and wider system issues such as a lack of guidelines and funding.^27^ Based on the evidence and implementation frameworks,^28^ recommendations were made for developers, primary care teams, and policymakers (Figure 1).

However, the review did not stratify results according to health conditions and it is therefore unknown if these recommendations are applicable across CDSSs. Implementation of cancer-specific CDSSs might differ from CDSSs targeting other conditions because of a number of factors, including the perceived severity of cancer, comparatively low incidence in primary care, the existence of screening programmes, and the wide range of potential symptoms. Barriers to CDSS implementation may also differ depending on its function in the diagnostic pathway, for example, risk assessment after symptomatic presentation versus shared decision making for screening participation.

The aim of this study was to determine whether similar recommendations apply for cancer-specific CDSSs, and how they can best be used by PCPs. Barriers to implementation of cancer-specific CDSSs in primary care were therefore examined and those for decision making in cancer screening compared with those for symptomatic presentation.

## Method

This analysis is a subanalysis of cancer-specific CDSSs from a recent systematic review on the implementation of CDSSs for the detection of disease in primary care,^27^ and is reported in line with the PRISMA 2020 statement. The initial review and the subanalysis of cancer-specific CDSSs compared with CDSSs for other conditions was registered using PROSPERO ( https://www.crd.york.ac.uk/PROSPERO/view/517054). However, after screening and full-text review, it was decided to differentiate between CDSSs for screening and those for symptomatic presentation, as they served different functions in the diagnostic process.

### Data sources and search strategy

With an experienced librarian, a search strategy was developed based on implementation frameworks^29,30^ and similar search strategies from reviews on barriers and facilitators of CDSSs in primary care.^31–33^ The original search included CDSS MeSH terms, terms around barriers and facilitators, primary care, and disease detection. Medline (through Ovid), Embase, Scopus, Web of Science, and Cochrane were searched on 5 October 2023 and the search was updated on 20 August 2024 (see Supplementary Information S1).

### Inclusion and exclusion criteria

Studies were included in the systematic review if they met prespecified PICOS-based criteria:

- Population: research involving primary care settings with PCPs, other staff, patients, or caregivers.
- Intervention: CDSS aimed to improve disease detection, broadly defined to cover screening, triage, risk assessment, test ordering, and referral recommendations. CDSS focused solely on prevention or future risk, or designed only for patients (for example, symptom checkers) were excluded.
- Comparison: not applicable.
- Outcomes: studies reporting barriers to implementing or using CDSSs, across both actual and hypothetical systems.
- Study type: primary research regardless of country. Reviews and abstracts without full text were excluded, although relevant reviews were used to identify additional studies.

### Procedure and analysis

Covidence was used for title and abstract screening, full-text review, and data extraction, all conducted independently by two authors. Discrepancies were resolved by discussion with a third reviewer if necessary. Interrater reliability was good for title and abstract screening (Cohen’s Kappa 0.88) and satisfactory for full-text review (Cohen’s Kappa 0.60). Discrepancies were discussed between reviewers and resolved by a third reviewer if necessary. Data from included studies were extracted using a coding sheet (see Supplementary Information S2).

Qualitative statements regarding barriers were extracted and assigned a summarising phrase. Quantitative findings were transformed (‘qualitised’) into textual descriptions and integrated with qualitative data as recommended by the Joanna Briggs Institute.^34^ All statements were analysed using a deductive-inductive framework approach supported by the conceptual underpinning of the Theoretical Domains Framework (TDF).^29^

The TDF was chosen as the domains cover a range of behavioural determinants, including individual skills, beliefs, and attitudes, as well as social and environmental influences, and is part of the Behaviour Change Wheel, and can be linked to interventions and policy recommendations targeting specific behavioural determinants.^28^

In a first step, summarising phrases were assigned to the data and mapped to TDF domains in a deductive approach by one author (the first author). Statements were coded into >1 domain if necessary. Following deductive coding, it became apparent that domains spanned various topics (for example, workflow disruptions, technical issues, and medicolegal concerns in the environmental domain). Therefore, a thematic analysis was applied as a second analysis step to inductively define themes within each TDF domain and therefore differentiate barriers to the implementation of CDSSs further. A second author (the senior author) checked the final coding, including the agreement of deductively coded themes into the TDF domains and the inductive development of sub-themes for clarity and coherence. The group of authors discussed the final framework.

For a deeper understanding of themes, two authors (the first and second authors) descriptively summarised statements within themes and chose representative quotes from included studies as examples.

Cancer-specific studies were categorised into CDSSs for:

- decision making around screening including prostate-specific antigen (PSA) testing in asymptomatic patients; and
- risk assessment and referral recommendations after symptomatic presentation to primary care.

Studies investigating PSA testing were added to the category for screening, as the included studies examined public perceptions of using PSA testing in asymptomatic populations, rather than as investigation for symptomatic patients. The number (and proportion) of studies mentioning barriers in these groups was counted and compared, and reported descriptively, with medium differences in the frequency of studies for cancer screening and symptomatic presentation reporting barriers over 20% and large differences over 30%. Thresholds were predefined pragmatically to exclude small differences that might have been caused by differences in study methodology between groups. The proportion of studies mentioning barriers was chosen as a metric to reduce the potential overinflation of barriers mentioned repeatedly in single studies, especially qualitative studies, and to account for differences in number of studies between groups.

To account for potential bias caused by differences in study methodology between groups, a sensitivity analysis was conducted restricted to purely qualitative studies.

### Risk of bias assessment

Two reviewers independently conducted quality appraisals using the Quality Assessment with Diverse Studies.^35^ The tool allows for narrative analysis of the risk of bias across 13 different criteria, including conceptual underpinning, description of setting, data collection, and analysis, as well as quality of study design. Criteria are rated on a scale from zero (no mention at all) to three (complete).

### Patient and public involvement

Two patient and public representatives were involved in the systematic review and commented on the manuscript. They took part in project meetings in which the systematic review was discussed. After conducting interim analyses, the authors discussed the themes to sense check the interpretation and consider implications from a patient perspective.

## Results

### Description of included research studies and CDSSs

**Figure 2.**
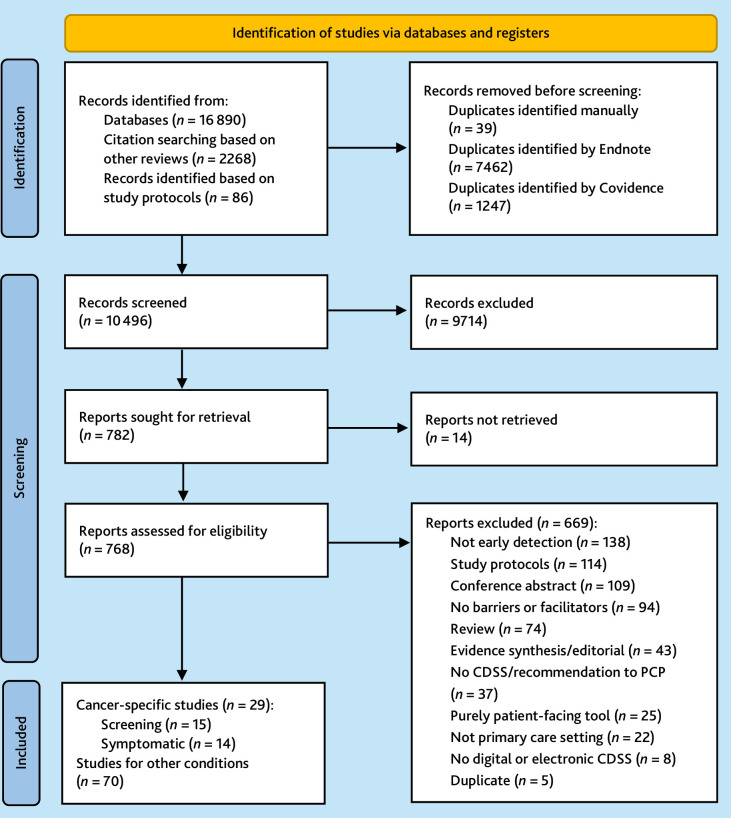
Flowchart. CDSS = clinical decision support system. PCP = primary care practitioner.

The review included 99 studies overall, with 70 studies investigating CDSSs for non-cancer conditions^27^ and 29 studies addressing cancer-specific CDSSs (Figure 2; see also Supplementary Table S1 for an overview of cancer-specific CDSSs and Supplementary Information S3, which includes full references for all 99 studies).

The 29 studies addressing cancer-specific CDSSs included 15 studies describing 10 different CDSSs for cancer screening^15–17,36–47^ and 14 studies describing 12 different CDSSs for symptomatic presentation.^18–20,26,48–57^

The geographical distribution of studies, and therefore healthcare system, overlapped strongly with CDSS function. Most studies on CDSSs for screening (*n* = 13/15, 87%) were conducted in the US,^15–17,36,37,40–47^ with two studies on CDSSs for screening conducted in Australia and Belgium.^38,39^ Most studies on symptomatic presentation (*n* = 8/14, 57%) were conducted in the UK,^18,19,26,49,52–54,57^ where no studies regarding screening were conducted. The other six studies on symptomatic presentation were conducted in Australia, Sweden, Brazil, and the US.^20,48,50,51,55,56^

**Figure 3.**
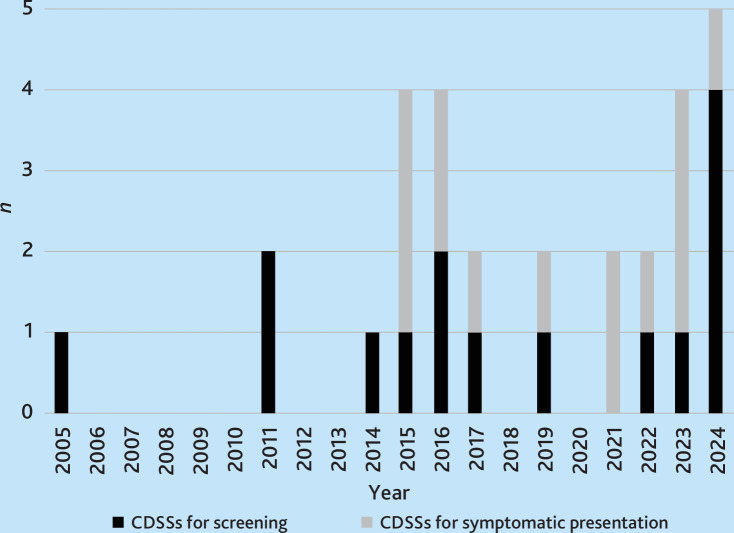
Year of publication of CDSSs for screening and symptomatic presentation. CDSS = clinical decision support system.

Studies were published between 2005 and 2024, with most studies published after 2014. Figure 3 provides an overview of year of publication depending on type of CDSS.

Most studies (*n* = 12/14, 86%) investigating CDSSs for symptomatic presentation^18–20,26,48–50,52–55,57^ and about half of the studies on CDSSs for screening^15,38,39,41,42,44–46^ used qualitative methods.

For studies examining CDSSs for symptomatic presentation, there was a range of functions of the CDSS. For example, of the 14 studies, eight studies (57%) facilitated referral,^18,19,26,48,49,52,54,57^ five (36%) conducted risk assessments,^50,51,53,55,56^ and three (21%) described CDSSs that gave management recommendations.^19,20,54^

Three (20%) of the 15 studies on screening investigated hypothetical CDSSs,^40,42,43^ 10 (67%) explored CDSSs used in pilot studies or larger trials,^15,17,36–39,44–47^ and two (13%) explored CDSSs for routine use.^16,41^ For the 14 studies on symptomatic CDSSs, three (21%) investigated hypothetical CDSSs,^50,54,56^ eight (57%) explored CDSSs used in pilot studies or larger trials,^20,26,48,49,51,53,55,57^ and three (21%) explored CDSSs for routine use.^18,19,52^

**Figure 4.**
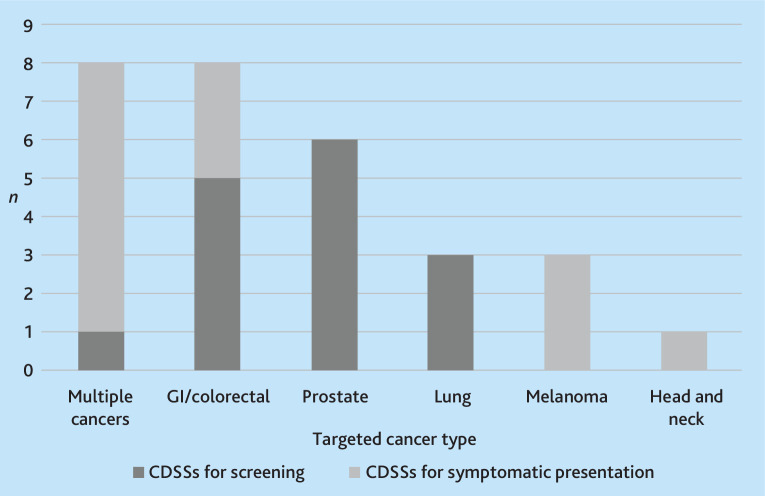
Targeted cancer types of CDSS for screening and symptomatic presentation. CDSS = clinical decision support system. GI = gastrointestinal.

See Figure 4 for an overview of targeted cancers.

### Risk of bias assessment

The justification of analytic methods, use of theoretical or conceptual underpinning, and stakeholder involvement were rated as very limited to basic, thus being prone to bias. Appropriateness of method of analysis, clarity of rationale and description of data collection, critical discussion of strengths and limitations, provision of recruitment data, as well as appropriateness of sampling to address research aims, were mostly rated as basic/moderate (see risk of bias assessment in Supplementary Information S4).

### Barriers to implementation

#### Comparison between CDSSs for cancer and for other conditions

In total, 328 statements of barriers in the 29 studies of cancer-specific CDSSs and 824 statements in the 70 studies that investigated CDSSs for other conditions were identified. There were minimal differences between the types of barriers reported for cancer-specific CDSS (overall) and those for CDSSs for other conditions (see Supplementary Table S2 for comparison of barriers for CDSSs for other conditions and cancer-specific CDSSs).

#### Comparison between cancer-specific CDSSs for screening and symptomatic presentation

In total, the current study identified 136 statements of barriers to implementation in studies on CDSSs for cancer screening (*n* = 15, predominantly from the US) and 192 statements in studies on CDSSs for symptomatic presentation (*n* = 14, predominantly from the UK and Australia; see Supplementary Table S3 and Supplementary Figure S1 for a forest plot of confidence intervals). Barriers are described with examples in Supplementary Table S4 (CDSSs for screening) and Supplementary Table S5 (CDSSs for symptomatic presentation).

The largest differences in reported barriers to implementation referred to knowledge and skills (*n* = 7/14, 50%, of symptomatic studies versus *n* = 2/15, 13%, of screening studies citing barriers in these domains). The domains not only referred to technical knowledge and skills needed to use new systems, but also medical knowledge to manage and follow-up on cancer risk, as well as a potential risk of deskilling if CDSSs are over-used:

*‘I gather from Future Health Today I should be actively looking for occult cancers in this group. But I need — I need a bit more education about it.’* (GP)^20^

*‘I think the downside of … making everything a tick-box exercise, does take away your clinical judgment, you can de-skill.’* (Practitioner)^52^

Studies on CDSSs for symptomatic presentation also reported barriers regarding negative emotions more frequently (*n* = 8/14, 57%, of symptomatic studies versus *n* = 4/15, 27%, of screening studies), driven by CDSSs potentially causing anxiety if cancer is suddenly suspected (*n* = 4/14, 29%, of symptomatic studies versus *n* = 0/15, 0%, of screening studies), and providers’ scepticism (*n* = 4/14, 29%, of symptomatic studies versus *n* = 0/15, 0%, of screening studies):

*‘“Wow! Listen, you got a 38% chance of cancer.” General public are very — I would find that confronting and the general public have a shocking record at understanding risk.’* (GP)^48^

*‘... you know initially I was very sceptical about this tool.’* (GP)^18^

Similarly, studies on symptomatic presentation were more likely to report barriers connected to beliefs that CDSSs would interfere with decision-making processes (*n* = 6/14, 43%, of symptomatic studies versus *n* = 2/15, 13%, of screening studies) and concerns around medicolegal consequences and liability (*n* = 4/14, 29%, of symptomatic studies versus *n* = 0/15, 0%, of screening studies):

*‘I personally don’t think tools help me too much, interfere with my process of thought, would have done better without it, it was unnatural.’* (GP)^53^

*‘Quite a few partners were worried about any medico-legal implications with that* … *if patients knew that you had a list of them with the risk and you hadn’t acted on it, what would be the implications?’* (GP)^49^

*‘It would take quite a lot of evidence that this is accurate. The question is what happens if I free someone from a melanoma based on the tool, am I responsible for it or the person who developed this app?’* (PCP)^51^

Barriers concerned with professional roles in primary care and interference with workflow were also more often cited in studies of CDSSs for symptomatic presentation. For example, there was a lack of integration of CDSSs into workflow and care processes (*n* = 9/14, 64%, of symptomatic studies versus *n* = 4/15, 27%, of screening studies), beliefs that providers would be more capable to complete tasks without the CDSS (*n* = 5/14, 36%, of symptomatic studies versus *n* = 1/15, 7%, of screening studies), and a low flexibility of CDSSs to allow for an individual approach (*n* = 3/14, 21%, of symptomatic studies versus *n* = 0/15, 0%, of screening studies):

*‘Because here we’ve had to keep a dual system approach, the admin team just are too stretched* […] *it means that I kind of hold the ball for not only my own two week wait referrals but you know for all the other two week wait referrals that are made in the practice via* [E-SN software]*.’* (GP)^19^

*‘I might do the recalls, I might call people in but in the end, GPs need to be accountable for their work and not me.’* (Practice nurse)^20^

Finally, a lack of stakeholder involvement and effective communication in the design or implementation was more common with three (21%) of 14 symptomatic studies versus none (0%) of the 15 screening studies.

On the other hand, studies on CDSSs for screening were more likely to report barriers concerning the negative effects of CDSSs on the patient–provider relationship (*n* = 2/14, 14%, of symptomatic studies versus *n* = 6/15, 40%, of screening studies) and general low willingness to use technological advances in primary care (*n* = 0/14, 0%, of symptomatic studies versus *n* = 3/15, 20%, of screening studies):

*‘*[I’m] *in favor of tool usage, but concerned about perceived benefits of screening and how to communicate screening.’* (Primary care clinician)^46^

Studies on CDSSs for screening also more frequently reported a lack of coherent way to track, monitor, and follow-up on patients (*n* = 1/14, 7%, of symptomatic studies versus *n* = 4/15, 27%, of screening studies):

*‘... sometimes you just have to use your best guess* [about which patient the document pertains to].*’* (Primary care clinician)^41^

The sensitivity analysis restricted to qualitative studies revealed a similar pattern of results (see Supplementary Table S6). The qualitative studies highlighted further differences between CDSSs for symptomatic presentation and CDSSs for screening with regard to confusion about benefits and purpose, time constraints, and beliefs regarding patients’ capability to use CDSSs.

## Discussion

### Summary

This systematic review analysed barriers to the implementation of CDSSs for cancer detection to establish whether general recommendations for the design and implementation of CDSSs in primary care are as applicable to CDSSs specifically aimed at early cancer detection. Barriers were reported with similar frequencies for cancer-specific CDSSs and CDSSs for other conditions, indicating that the recommendations presented in Figure 1 and Derksen *et al* ^27^ remain relevant across conditions. CDSSs were not used because of time constraints, lack of integration into systems and workflows, low usability and cognitive overload, fears around adverse effects on decision making and patient outcomes, confusion about benefits and purposes, lack of trust in CDSS results, need for training, frustration caused by technology, and a negative impact on the patient–provider relationship.

There were a number of differences between cancer-specific CDSSs for symptomatic presentation and those for cancer screening, showing that the aim of the CDSS and the context in which it is to be used are crucial for implementation. A specific barrier that was more frequently reported in studies of CDSSs for symptomatic presentation included a more difficult integration into workflow and care processes. However, as studies of CDSSs for symptomatic presentation were more common in the UK and Australia, workflow integration challenges may reflect the organisation of primary care and use of national electronic health record (EHR) systems in these countries. Other common challenges related to CDSSs for symptomatic presentation in these contexts included medicolegal uncertainty, requirement of more skills and knowledge, higher potential to interfere with decision-making processes, and more negative emotions of practitioners and patients. However, when looking at qualitative studies only, the belief that patients might not be sufficiently capable to use CDSSs was more common in studies on CDSSs for screening.

### Strengths and limitations

This systematic review allowed the comparison of both condition-specific and function-specific implementation factors. Use of the TDF, a well-established implementation framework, supported the synthesis of a broad range of themes. However, the smaller number of cancer-specific studies (*n* = 29) precluded statistical comparison of reported barriers, and confidence intervals are overlapping even where large descriptive differences in proportions were observed. Hence, results should be interpreted with caution, and research approaches directly comparing cancer-specific CDSSs for different functions are warranted.

Methodological differences also affected comparability as more studies on CDSSs for symptomatic presentation than for screening used qualitative methods. This likely contributed to the greater number of barriers reported for symptomatic CDSSs.

A key limitation is that it was not possible to disentangle the influence of the healthcare system from CDSS function. Most screening studies were US-based (87%, *n* = 13/15), whereas the majority of symptomatic studies were conducted in the UK and Australia, meaning that the observed differences between barriers regarding these two types of CDSS (for example, medicolegal uncertainty and workflow integration) are likely to reflect contextual rather than functional factors. In terms of the quality of included studies, many studies lacked a theoretical foundation, potentially influencing which barriers were explored.

### Comparison with existing literature

Considering the context of the diagnostic process, it is not surprising that fewer complexities were reported in studies of CDSSs for cancer screening as these are often designed to support shared decision making regarding the patient’s screening attendance by improving understanding of benefits and risks of screening.^58^ Templates integrated in CDSSs can support clinicians in providing standardised, acceptable information supporting shared decision making (for example, Engelen *et al* and Morgan *et al*).^39,42^

Ruling in or out cancer, especially given the variability of (non-specific) presentations and complex diagnostic processes in primary care, demands more complex CDSSs.^59,60^ PCPs need to consider potential alternative explanations, which may involve eliciting more information, ordering tests, investigations, or referrals.^61^ Accordingly, clinicians reported concerns that CDSSs might have adverse effects and interfere with their diagnostic processes.^48,53^ Clinicians were also concerned about legal ramifications if cancer is missed after a low-risk assessment,^49,52^ or feared that mentioning the possibility of cancer early in the diagnostic process would cause unnecessary patient distress.^18,20,48^

### Implications for research and practice

The findings of this review suggest that factors related to the specific function of the diagnostic process, clinicians, and healthcare system may be more important for implementation success than the specific condition targeted by a CDSS. Small differences between barriers of CDSSs for cancer and other conditions indicate that recommendations to address common barriers are relevant irrespective of the target condition, including system integration, ensuring usability, supporting adaptability, and carefully testing and evaluating CDSSs.

Implementation of CDSSs for symptomatic presentation needs careful attention to ensure success. The current findings mirror previous research showing that high workload and poor workflow integration hinder CDSS uptake, especially during consultations (for example, Fletcher *et al* and Meunier *et al*).^32,33^ PCPs should be actively involved in CDSS development to increase understanding of current practice and decision-making processes, as well as recurring diagnostic challenges. However, few salient benefits or rewards are provided for PCPs getting involved in development and testing of CDSSs in primary care.

There is also a need to support monitoring and decision making in the post-consultation space, such as through effective safety netting.^62–64^ Primary care teams need to assign responsibility to team members so that follow-up actions emerging from additional tests or persistent and changing symptoms (for example, monitoring and patient communication) are addressed, while limiting burden to clinicians.

CDSSs for symptomatic presentation require more knowledge and skills both regarding the use of new technologies, as well as the detection and management of cancer.^18,20^ PCPs should therefore incorporate training on EHR systems and technologies, as well as cancer diagnosis and referral, in their practice. This can help to evaluate new CDSSs and their potential impact before and during implementation,^14^ including AI-based CDSSs.^65,66^ As AI technologies pose risks including bias in algorithms potentially affecting CDSS accuracy and concerns about data privacy,^67^ it is even more critical for primary care practices to ensure regulation and quality-control mechanisms, as well as know about insurance and liability policies.

Addressing barriers related to medicolegal issues and trust is especially important in AI-based CDSSs. Primary care practices need to comply with data protection regulations for all data used for AI-based decision-support to maintain public trust. For example, practices need to confirm that AI-based CDSSs meet medical device standards and clarify professional liability.^68,69^ Training regarding AI literacy and data governance should be part of PCPs’ professional development to ensure adequate use in primary care.^70^ As AI algorithms are more complex than conventional CDSS risk prediction models, interpretability of results and recommendations can be more difficult. Hence, more effort is needed to improve interpretability of predictions.^71^ Although this challenge needs to be addressed by policymakers and CDSS developers, primary care teams can prioritise CDSSs that provide understandable and explainable recommendations that can be communicated to patients.^72^

However, PCPs need to be supported by both developers who can ensure CDSS adaptability to changing guidelines, as well as commissioners and policymakers who need to provide guidance on evidence-based CDSSs, including data security and medicolegal practice.

The generalisability of these recommendations across low- and middle-income countries (LMICs) is limited as all but one of the studies investigating cancer-specific CDSSs were from high-income countries. Saraiva *et al* ^55^ investigated a CDSS for symptomatic presentation in Brazil. However, the focus was on model validation rather than implementation, and barriers were only briefly described. However, CDSS implementation in LMICs might face more difficulties because of a lack of technical infrastructure (for example, Cano *et al* and Tewari *et al*),^73,74^ lower awareness of cancer and acceptability of screening programmes,^75,76^ and generally limited healthcare resources.^27^ If technological advances are exclusively developed and implemented in high-income countries, researchers and policymakers risk widening inequalities in early cancer detection and therefore survival globally. Hence, research and resource allocation through global programmes is needed to dissect setting-specific barriers in LMICs.

In conclusion, this review reports few differences between CDSSs for different conditions, but more pronounced differences in proportions of studies reporting barriers to implementation for cancer-specific CDSSs for screening and symptomatic presentation. This indicates that recommendations for the implementation of CDSSs are relevant across conditions but should be adapted to the aim and context of a CDSS. There is a need to consider how CDSSs can support diagnostic decision making in primary care in patients with unclear or challenging cases, especially in the light of emerging technologies such as AI.

## Data

The datasets generated and/or analysed during the current study are available in the Open Science Framework repository (https://osf.io/qp3y6).
